# Cancer Incidence in Physicians: A Taiwan National Population-based Cohort Study

**DOI:** 10.1097/MD.0000000000002079

**Published:** 2015-10-30

**Authors:** Yu-Sung Lee, Chien-Chin Hsu, Shih-Feng Weng, Hung-Jung Lin, Jhi-Joung Wang, Shih-Bin Su, Chien-Cheng Huang, How-Ran Guo

**Affiliations:** From the Department of Emergency Medicine, Chi-Mei Medical Center (Y-SL, C-CH, H-JL, C-CH); Department of Biotechnology, Southern Taiwan University of Science and Technology, Tainan (C-CH, H-JL); Department of Healthcare Administration and Medical Informatics, Kaohsiung Medical University, Kaohsiung (S-FW); Department of Emergency Medicine, Taipei Medical University, Taipei (H-JL); Department of Medical Research, Chi-Mei Medical Center (J-JW); Department of Leisure, Recreation and Tourism Management, Southern Taiwan University of Science and Technology (S-BS); Department of Occupational Medicine, Chi-Mei Medical Center (S-BS, C-CH); Department of Medical Research, Chi-Mei Medical Center, Liouying (S-BS); Department of Environmental and Occupational Health, College of Medicine, National Cheng Kung University (C-CH, H-RG); Department of Child Care and Education, Southern Taiwan University of Science and Technology (C-CH); Department of Geriatrics and Gerontology, Chi-Mei Medical Center (C-CH); and Department of Occupational and Environmental Medicine, National Cheng Kung University Hospital, Tainan, Taiwan (H-RG).

## Abstract

Cancer has been the leading cause of death in Taiwan since 1982. Physicians have many health-related risk factors which may contribute to cancer, such as rotating night shift, radiation, poor lifestyle, and higher exposure risk to infection and potential carcinogenic drugs. However, the cancer risk in physicians is not clear. In Taiwan's National Health Insurance Research Database, we identified 14,889 physicians as the study cohort and randomly selected 29,778 nonmedical staff patients as the comparison cohort for this national population-based cohort study. Cox proportional-hazard regression was used to compare the cancer risk between physicians and comparisons. Physician subgroups were also analyzed. Physicians had a lower all-cancer risk than did the comparisons (hazard ratio [HR] 0.86, 95% confidence interval [CI] 0.76–0.97). In the sex-based analysis, male physicians had a lower all-cancer risk than did male comparisons (HR 0.82, 95% CI 0.73–0.94); and female physicians did not (HR 1.29, 95% CI 0.88–1.91). In the cancer-type analysis, male physicians had a higher risk of prostate cancer (HR 1.72, 95% CI 1.12–2.65) and female physicians had twice the risk of breast cancer (HR 2.00, 95% CI 1.11–3.62) than did comparisons. Cancer risk was not significantly associated with physician specialties. Physicians in Taiwan had a lower all-cancer risk but higher risks for prostate and breast cancer than did the general population. These new epidemiological findings require additional study to clarify possible mechanisms.

## INTRODUCTION

Cancer is the leading cause of death in economically developed countries, and the second leading cause of death in developing countries.^1^ In Taiwan, cancer has been the most common cause of death since 1982.^2^ In 2013, cancer had a standardized mortality of 130.4 people per 100,000 population and contributed 29.0% of all deaths, which is significantly higher than the second cause of death, cardiovascular disease, which contributed 11.5%.^2^

Prevention offers the most cost-effective long-term strategy for controlling cancer because at least one-third of all cancer cases are preventable.^3^ The World Health Organization proposed several preventable risk factors: smoking, chewing, and snuffing tobacco; physical inactivity, dietary factors, obesity, and overweight; alcohol drinking; infections; environmental pollution; occupational carcinogens; and radiation.^3^

Physicians have many health-related risk factors which may contribute to cancer. Physicians are more likely than other healthcare workers to have close contact with patients; therefore, they are more vulnerable to infection.^4^ After the initiation of National Health Insurance (NHI) in 1995, Taiwanese have had greater access to health care,^5^ which has increased the workload for physicians. Almost half of Taiwanese physicians work more than 57 hours/week, 34.5% work as many as 65 hours/week, and 10.6% need an average of 21 extra work hours for morning meetings, academic research, and teaching.^6^ Physicians, especially emergency and critical care specialists, have to take rotating night shifts (RNS), which is also suggested to be a risk factor for cancer.^7–10^ Insufficient time to maintain a life style that includes adequate physical activity and a healthy diet are also major problems.^6,11^ In addition, physicians are potentially exposed to several suspected hazards such as X-rays, anesthesia gases, chemotherapy drugs, antiviral drugs, and sterilizing agents.^12–15^ However, there are insufficient studies about physician cancer. Therefore, we wanted to investigate whether physicians in Taiwan have a higher risk of cancer than does the general population.

## METHODS

### Data Sources

Taiwan's NHI program covers all citizens except prison inmates. The NHI Research Database (NHIRD), one of the largest and most comprehensive databases of its type in the world, covers 99% of the inpatient and outpatient claims for Taiwan's population of more than 23.3 million.^16–18^ The NHIRD contains encrypted patient identification numbers, International Classification of Diseases, Ninth Revision, Clinical Modification (ICD-9-CM) codes for basic sociodemographic information, including sex and date of birth, dates of admission and discharge, clinical diagnoses and procedures, and prescribed medications.^18^ Information on medical personnel (including physicians, nurses, pharmacists, and other healthcare providers) is also available and includes specialty, date licensed, work area, hospital level, types of employment, and encrypted identification number, which can be linked to the aforementioned claims data.^18^ The NHI covers all the expenses of diabetes mellitus (DM), hypertension (HTN), hyperlipidemia, coronary artery disease (CAD), and cancer.^18^ Despite the above fact, we did not have complete information on the physicians’ history of employment including loss of follow-up and resignation, which also limited causal inferences between one's profession and the risk of cancer. Also, the participant in the comparison cohort may turn to be a physician. However, these limitations would not affect the final result because of the large physician and comparison cohort.^4^

### Ethics Statement

This study was conceived in line with the Declaration of Helsinki and was approved by the Institutional Review Board at Chi-Mei Medical Center. Informed consents from the patients were waived because the dataset used in this study consists of de-identified patient data released to the public for research. The rights and welfare of the patients were not affected by the waiver of informed consent.

### Definition of the Characteristics

We used 35 years as a cut-off point of age because biological function and physical performance reach their peak at 35 years of age.^19^ Income was defined as low (monthly income <new Taiwan dollar [NT$] 15,840), medium (monthly income NT$ 15,840–25,000), and high (monthly income >NT$ 25,000) defined by insurance premium.^20^ Residence location was defined as north, center, south, and east according to Taiwan's administrative regions. Level of hospital was defined as medical center, regional hospital, community hospital, and local clinic according to the criteria by Taiwan's Ministry of Health and Welfare.^21^

### Physicians and Comparisons (General Population): Selection and Analysis

**FIGURE 1:**
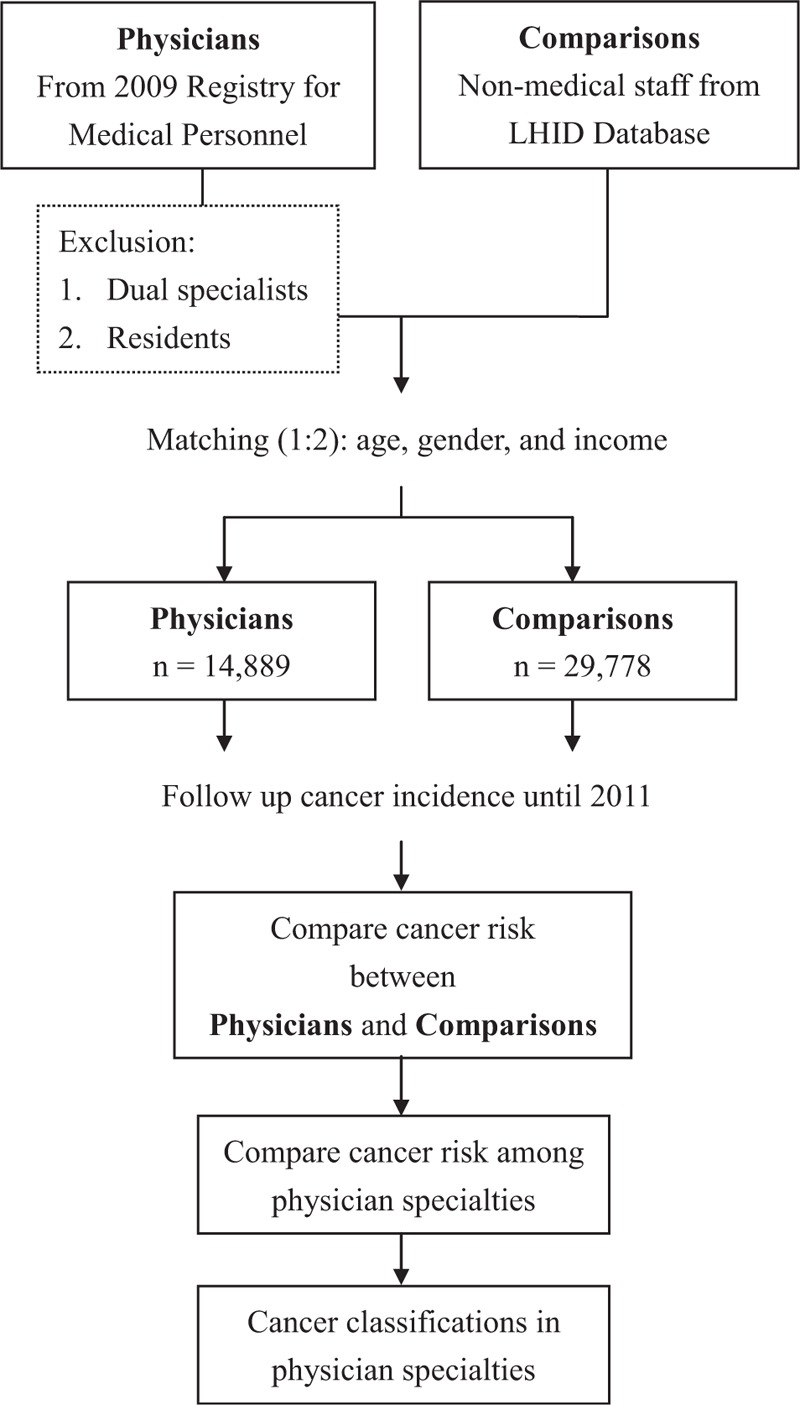
Flow chart for the study. LHID = Longitudinal Health Insurance Database.

Data of the physicians were obtained from the Registry of Medical Personnel, which contains a record of all registered medical staff in 2009. We then excluded physicians who were residents and dual specialists (eg, a physician board-certified in surgery and emergency medicine). We excluded dual specialists because of the difficulty of assigning them to a specific subgroup for comparison. We also excluded residents because their practice time in individual specialties is short. In the comparison cohort (general population), we selected 2 nonmedical staff matches per case from the Longitudinal Health Insurance Database 2000 (LHID2000), which contains all claims data of one million (4.34% of the population) beneficiaries who were randomly selected in 2000. There are no significant differences in healthcare costs, age, and sex between all NHI enrollees and those in the LHID2000. Comparisons were matched with physicians by age, sex, and income (Fig. 1, Table 1). We matched age, sex, and income because they are related to cancer incidence, which may affect the baseline difference. Income is related to cancer by affecting lifestyle, accessibility to health-related social resources, and preventive medical checkups.^22,23^ A Statistical Analysis System macro “gmatch”^24^ was applied, which used a greedy-matching algorithm to select the nearest control without replacement. Both age and income were matched by treating continuous variables in the matching process.

**TABLE 1:**
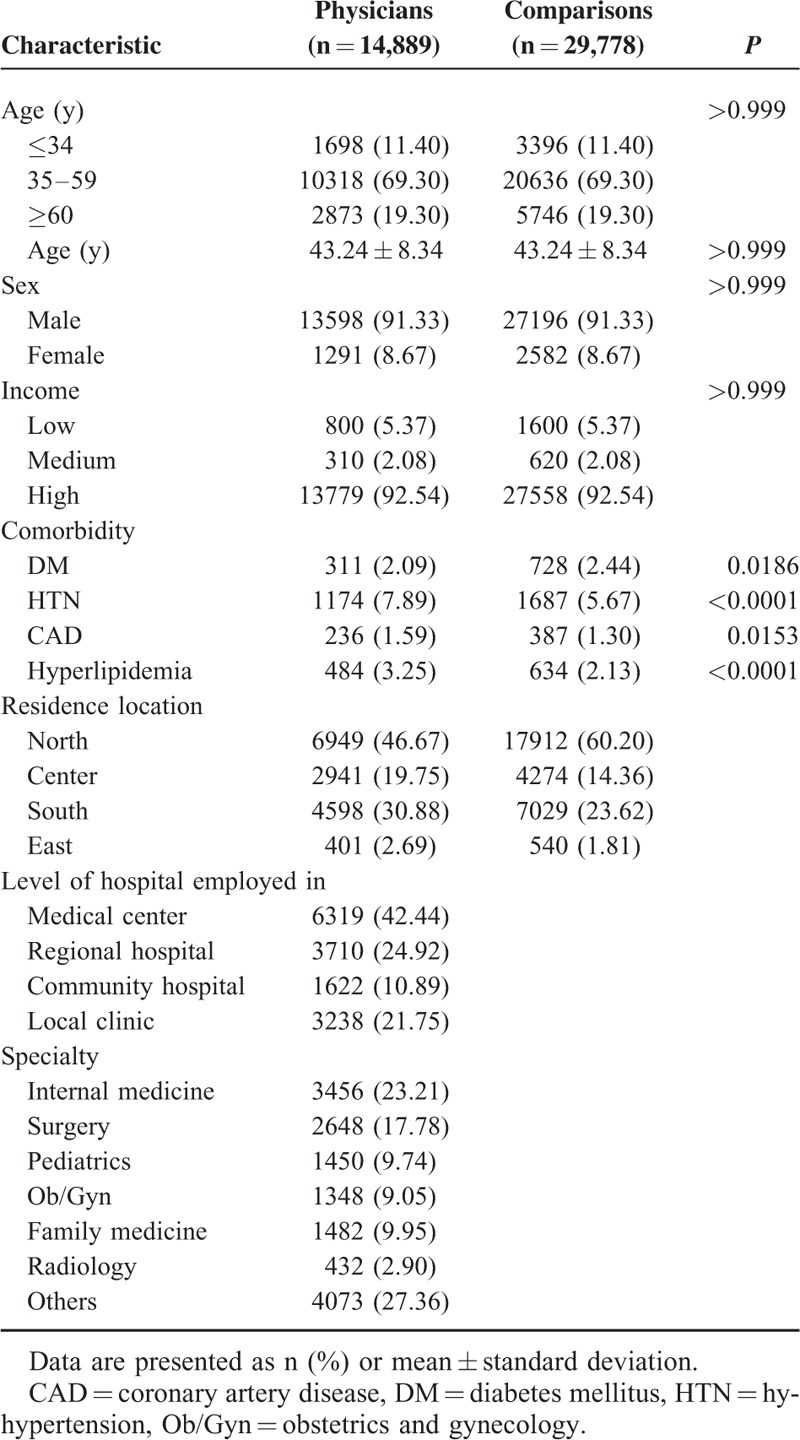
Demographic Characteristics and Comorbidities for Physicians and Comparisons

We linked to the diagnostic codes through the inpatient and ambulatory care claims databases of the NHI. Common comorbidities were DM (ICD-9 code 250), HTN (ICD-9 codes 401–405), hyperlipidemia (ICD-9 code 272), and CAD (ICD-9 codes 410–414.02). These 4 comorbidities were counted if they were diagnosed in 3 or more ambulatory care claims coded 12 months before the January 1, 2002 (index medical care date). Patients who had cancer before 2002 were excluded.

We compared the cancer risk between physicians and comparisons by following up their medical histories until 2011 (Fig. 1). Cancer was identified using a computerized algorithm that included the ICD-9 codes of 140–208.

### Physician Subgroup Analysis

We analyzed the subgroups of physicians for hospital level and specialty (Fig. 1). We felt that emergency and critical care specialists (internal medicine, surgery, obstetrics and gynecology, pediatrics) may have repetitive nerve stimulation and a less than healthy quality of life because of overwork, which may contribute to a higher risk for cancer. Radiologists exposed to ionizing radiation are also suggested to have a higher risk for cancer.^25^ Therefore, we divided physicians into 6 subgroups for comparison: internal medicine, surgery, obstetrics and gynecology, pediatrics, family, radiology, and others (eg, rehabilitation, psychology, dermatology, etc). The cancer classifications of individual specialties were also done.

### Statistical Analyses

Differences in baseline characteristics and comorbid variables between the 2 groups were evaluated using Student *t* test for continuous variables and Pearson chi-square test for categorical variables. We used Cox proportional-hazard regression to compute all the cancers and the subtype risks between the 2 cohorts. We also used Cox proportional-hazard regression to assess the risk of cancer between physicians and their comparisons stratified by age, level of hospital employed in, and specialty. For the physician subgroup analysis, Cox proportional-hazard regression with adjustment of age and sex was also used to explore the cancer risk among the physician specialties. Kaplan–Meier method was used to calculate the cumulative incidence rates of cancer in both cohorts, and the log-rank test was used to analyze differences between the 2 cohorts. SAS 9.3.1 for Windows (SAS Institute, Cary, NC) was used for all analyses. Significance was set at *P* <0.05 (2-tailed).

## RESULTS

### Basic Characteristics of Patients

We recruited 14,889 physicians and 29,778 age, sex, and income-matched comparisons (general population) (Table 1). The median age of the physicians was 43.24 ± 8.34 years. The proportion of <34-year-olds was 11.40%, of 35 to 59-year-olds was 69.30%, and of ≥60-year-olds was 19.30%. Most physicians were men (91.33%) and worked in a medical center (42.44%). Significantly more physicians than comparisons had HTN (7.89% vs 5.67%), CAD (1.59% vs 1.30%), and hyperlipidemia (3.25% vs 2.13%), but fewer had DM (2.09% vs 2.44%). There were 3456 (23.21%) physicians with specialties in internal medicine, 2648 (17.78%) in surgery, 1450 (9.74%) in pediatrics, 1348 (9.05%) in obstetrics and gynecology (Ob/Gyn), 1482 (9.95%) in family medicine, 432 (2.90%) in radiology, and 4073 (27.36%) in others (Table 1).

### Comparison of Cancer Risk Between Physicians and Comparisons and Subgroup Analyses of Physicians

**FIGURE 2:**
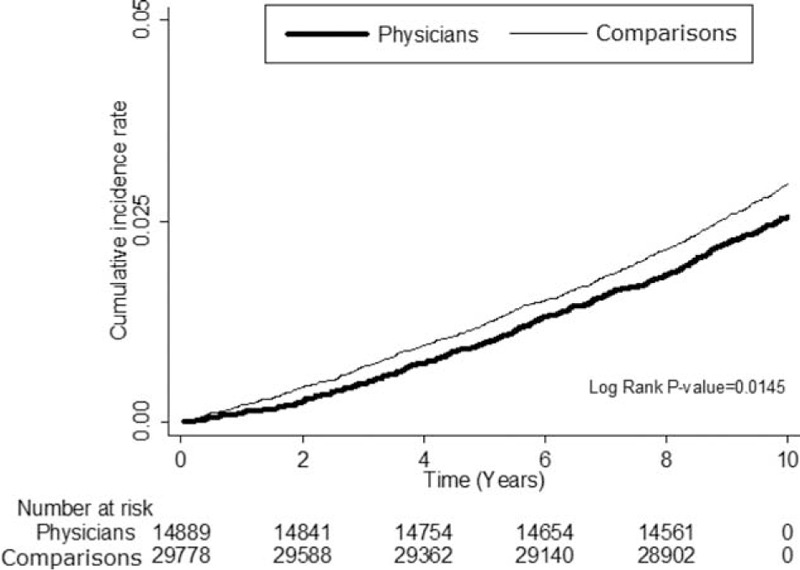
Cumulative incidence rate for cancer in physicians and comparisons during the follow-up.

In total, 2.51% of the physicians and 2.90% of the comparisons developed cancer and were followed up until 2011 (Table 2). Physicians had a significantly lower cancer risk than did the comparisons (hazard ratio [HR] 0.86, 95% confidence interval [CI] 0.76–0.97) (Table 2). Kaplan–Meier method and log-rank tests also showed that the physicians had a significantly lower cancer risk than did comparisons during the follow-up period (Fig. 2). Physicians who were 35 to 59 years old had a lower cancer risk (HR 0.81, 95% CI 0.68–0.97) than did comparisons of the same age; however, physicians aged <34 years and ≥60 did not (Table 2). Male physicians had a lower cancer risk (HR 0.82, 95% CI 0.73–0.94) than did male comparisons, but female physicians did not (HR 1.29, 95% CI 0.88–1.91) (Table 2). In the analysis of individual cancer risk between physicians and comparisons, male physicians had a significantly higher risk of prostate cancer (HR 1.72, 95% CI 1.12–2.65) and female physicians had twice the risk of breast cancer (HR 2.00, 95% CI 1.11–3.62) than did comparisons (Table 3).

**TABLE 2:**
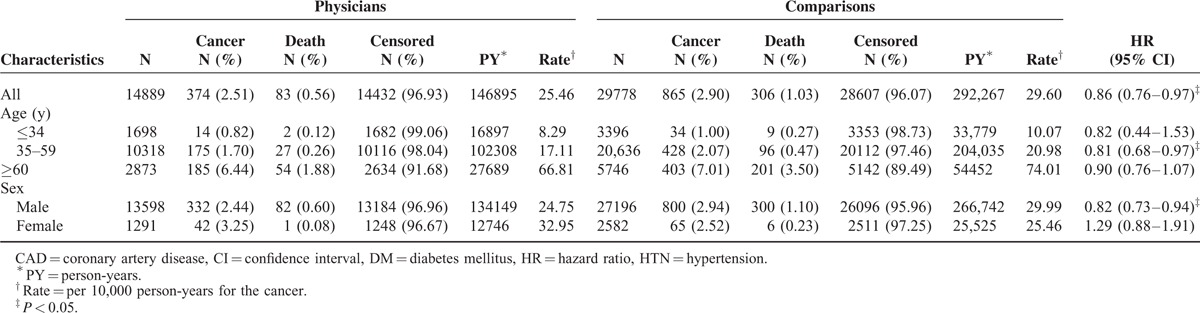
Comparison of All Cancer Risk Between Physicians and Comparisons by Cox Proportional-hazard Regression

**TABLE 3:**
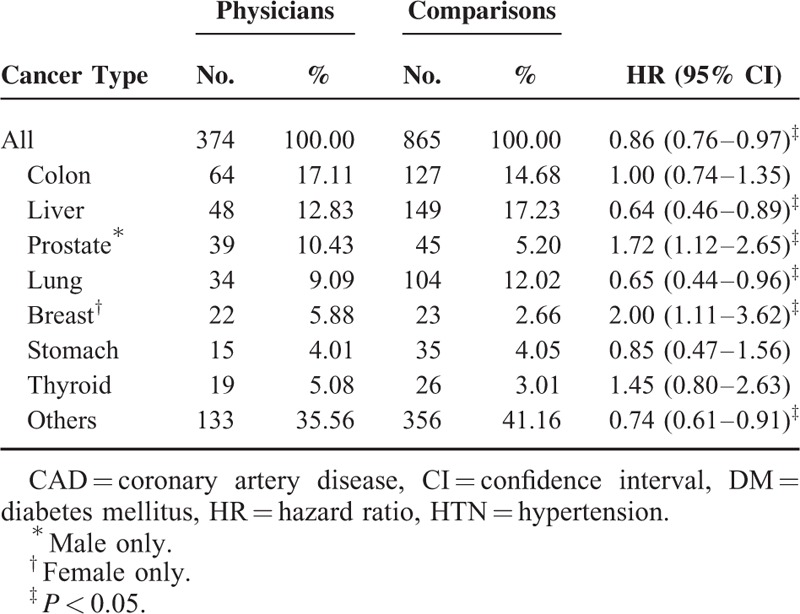
Comparison of Individual Cancer Risk Between Physicians and Comparisons by Cox Proportional-hazard Regression

Physicians employed in regional hospitals and local clinics had a lower all-cancer risk than did comparisons (adjusted HR [AHR] 0.75, 95% CI 0.58–0.98; and AHR 0.79, 95% CI 0.63–0.98, respectively); however, physicians employed in medical centers and community hospitals did not (Table 4). Although there were no significant differences in the cancer risk between the physician specialty subgroups and comparisons, radiologists tended to have a higher cancer risk (AHR 1.45, 95% CI 0.72–2.91) (Table 4) and compared with other physician specialists, a nonsignificantly higher cancer risk (AHR 1.40, 95% CI 0.79–2.49) (Table 5). Lung, colon, liver, prostate, and breast cancers were the most common types in physicians (Table 6). Radiologists had a higher percentage of lung cancer percentage (21.43%) than did other specialties.

**TABLE 4:**
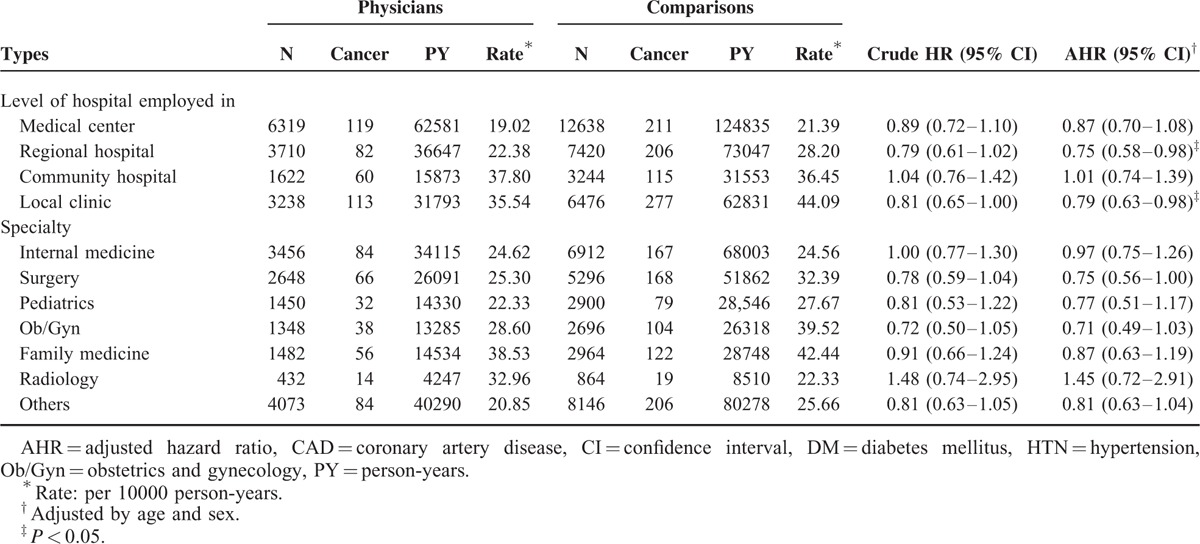
Comparison of All-cancer Risk Between Subgroup Physicians and Comparisons by Cox Proportional-hazard Regression

**TABLE 5:**
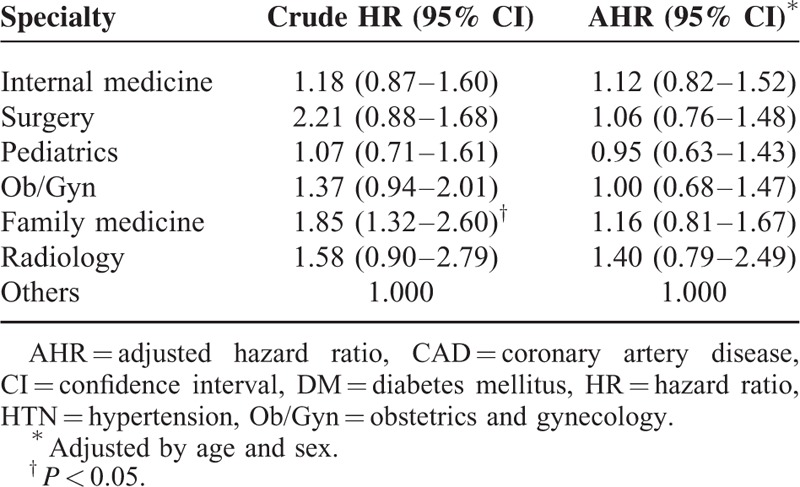
Comparison of All-cancer Risk Among Physician Specialties by Cox Proportional-hazard Regression

**TABLE 6:**
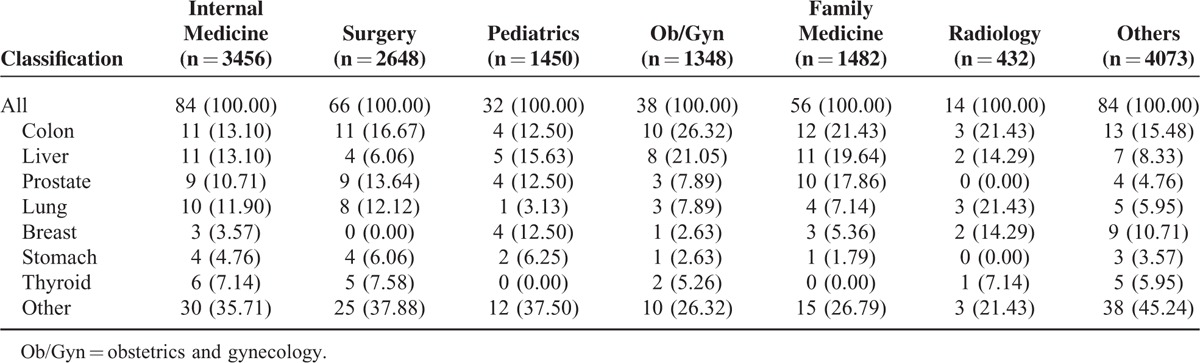
Cancer Classifications in Physician Specialties

## DISCUSSION

In this study of physician cancer, we found that physicians had a lower all-cancer risk than did the general population. In sex subgroup analysis, male physicians had a lower all-cancer risk than did male comparisons; however, female physicians did not have a lower risk than did female comparisons. Despite a lower all-cancer risk, male physicians had a higher prostate cancer risk and female physicians had a higher breast cancer risk than did the general population. Physician specialties related to emergency and critical care did not have a higher cancer risk than others. Radiologists tended to have a nonsignificantly higher all-cancer risk than did the general population and other medical specialists. The present study showed that the top 5 cancers in physicians were lung, liver, colon, prostate, and breast. It is similar to the ranking of cancer death in the general population of Taiwan: lung cancer; liver cancer; colon cancer; breast cancer; oral cancer; prostate cancer; gastric cancer; pancreatic cancer; esophageal cancer; and cervical and uterine cancer.^2^ Radiologists had a higher incidence of lung cancer than did other specialists. These findings suggested that despite a lower all-cancer risk, physicians are more likely than the general population to develop certain cancers. It is also a concern that radiologists tended to have a higher cancer risk than other specialties, especially lung cancer. From the results of this national population-based cohort study, we got a clearer picture of physician cancer. It provided us useful epidemiological information for future investigations of the underlying mechanism.

A possible mechanism for the higher rate of breast cancer in female physicians is the RNS.^26^ Despite no previous study about the association of RNS and breast cancer in physicians, there are many studies^26–28^ on night-shift work in general, and nurses in particular, which provide evidence of higher rates of breast cancer in women who work RNS. Female nurses who reported more than 20 years of RNS had a higher relative risk (RR) of breast cancer than did those who did not report any RNS (multivariate RR 1.79, 95% CI 1.06–3.01).^28^ Environmental lighting powerfully influences the circadian system in humans.^29^ RNS may have an adverse effect on breast cancer risk by suppressing melatonin, a hormone intimately linked to the circadian system and cancer-protective capability.^27,28,30^

Although there was no study solely about prostate cancer in physicians, RNS was suggested to be a risk factor for prostate as well as for breast cancer.^31,32^ For example, Kubo et al^31^ reported that the RRs of prostate cancer were 2.3 (95% CI 0.6–9.2) for fixed night work and 3.0 (95% CI 1.2–7.7) for RNS work. For Conlon et al,^32^ the odds ratio for prostate cancer was 1.19 (95% CI 1.00–1.42) for study participants who did full-time rotating shift work.

Both prostate and breast cancer are sex hormone-related cancers and, therefore, might have some common pathogenic factors. Other studies^33,34^ have reported that a history of prostate cancer in one or more first-degree relatives (father or brother) might also increase a woman's risk of breast cancer, especially if the prostate cancer was found at a young age.

Despite higher prostate and breast cancer risks, physicians had lower all-cancer risk than did the general population. The probable explanation is that physicians had better medical knowledge, higher disease awareness, and more economic resources, which may push them adopt healthy behaviors to compensate the risk factors from job.^4,17,35,36^ A recent study in Taiwan reported that physicians had a higher prevalence of HTN and hyperlipidemia, but a lower risk of acute myocardial infarction than did the general population.^35^ The authors concluded that physicians are not necessary healthier than the general population, but physicians have a greater awareness of disease and greater access to medical care, which permits timely treatment and may prevent critical conditions such as acute myocardial infarction induced by delayed treatment.^35^ Another study reported similar results about peptic ulcer disease in healthcare workers.^36^ Despite the long working hours, high job stress, and shift work, which are risk factors for peptic ulcer disease, physicians did not have a higher peptic ulcer disease risk than did the general population.^36^ The authors explained that physicians may have better coping skills and medical knowledge to manage their stress.^36^

Our study showed a higher all-cancer risk in radiologists than in the general population and other physician specialties, but it was not statistically significant. Radiologists were among the earliest occupational groups exposed to ionizing radiation from human-made sources.^25^ Because of high radiation exposure, early medical radiation workers had excess risks of breast, leukemia, and skin cancers.^25,37–39^ However, no excess cancer risk and mortality is evident among more recent medical radiation workers.^25,40–42^ Our study showed breast cancer and leukemia were 14.29% and 0% in radiologists with cancers, respectively. The reduction in occupational exposure and cancer risk may be due to the improvements in radiation protection practices recently.^25^ It is necessary to follow up these recent workers continuously because they are still young and exposed to new types of radiologic procedures.^25^

Except for RNS and radiation, there are few studies about other health-related risk factors for cancer in physicians. A study in 2012 showed that only 12.0% (23/191) of physicians had low lifestyle-related cancer risks, which were defined as not a current smoker, body mass index <28, regular recreational physical activity, and not consuming alcohol every day. Another study^43^ about workplace stressors and lifestyle-related cancer risk factors among female physicians also showed that only 13.4% had a low lifestyle-related cancer risk profile. However, the study provides no direct evidence about the relationship of health-related risk factors and physician cancer.

This study has some limitations. First, the number of cancer cases and a 10-year period (2002–2011) may not be enough. Additional studies of more cases and longer periods may be needed. Second, there was no information on the severity of the cancer, frequency of RNS, number of working hours, doses of radiation exposed to, levels of smoking, alcohol drinking, or betel nut chewing habits, severity of viral hepatitis, family history of cancer, or other risk factors for cancer; therefore, we were unable to evaluate these factors between physicians and comparisons. Collecting detail information and following for a long time for cancer incidence in a large physician cohort are really difficult at current stage. We tried to clarify this issue based on a nationwide population-based database by a scientific method. Despite the fact that it was not perfect, we believe this study provided us an insight for this issue and direction for subsequent research in the future. In addition, although we were able to adjust for the effects of these factors by taking into account health-related risk factors, further studies with direct measurements are helpful to evaluate the effects of these factors in physicians. Third, we excluded dual specialists which may cause a selection bias in this study. It would be of value to compare a single-specialty physician to those with dual specialties. Further study including patients with dual specialties as a special group is warranted. Finally, despite our study being national and population-based, our findings may not be generalizable to other countries.

## CONCLUSIONS

This is the first study to show that physicians have a lower all-cancer risk but higher prostate and breast cancer risks than does the general population. RNS may play a role, but other health-related risk factors could not be evaluated. Although the cancer risk for emergency medicine and critical care specialists was no higher than that for practitioners of other specialties, radiologists tended to have a nonsignificantly higher all-cancer risk than did the general population and other medical specialists, most likely because of their exposure to radiation. Additional investigations are needed to clarify this question.
